# A retrospective study on the combined biomarkers and ratios in serum and pleural fluid to distinguish the multiple types of pleural effusion

**DOI:** 10.1186/s12890-021-01459-w

**Published:** 2021-03-19

**Authors:** Liyan Lin, Shuguang Li, Qiao Xiong, Hui Wang

**Affiliations:** 1 Department of Clinical Laboratory, Peking University People’s Hospital, Xizhimen South Avenue No. 11, Beijing, 100044 China; 2 Institute of Medical Technology, Peking University Health Science Center, Beijing, 100191 China; 3 Department of Infectious Diseases and Immunology, Sydney Medical School, The University of Sydney, Sydney, 2006 Australia; 4 School of Public Health, The University of Sydney, Sydney, 2006 Australia

**Keywords:** Pleural effusion, Tuberculous pleural effusion, Biomarkers, Ratios, Differential diagnosis, Decision-tree analysis

## Abstract

**Purpose:**

Pleural effusion (PE) is a common clinical manifestation, and millions of people suffer from pleural disease. Herein, this retrospective study was performed to evaluate the biomarkers and ratios in serum and pleural fluid (PF) for the differential diagnosis of the multiple types of PE and search for a new diagnostic strategy for PE.

**Methods:**

In-patients, who developed tuberculous PE (TPE), malignant PE (MPE), complicated parapneumonic effusion (CPPE), uncomplicated PPE (UPPE), or PE caused by connective tissue diseases (CTDs) and underwent thoracentesis at Peking University People’s Hospital from November 2016 to April 2019, were included in this study. Eleven biomarkers and their ratios in serum and PF were investigated and compared between pairs of the different PE groups, and a decision-tree was developed.

**Results:**

Totally 112 PE cases, including 25 MPE, 33 TPE, 19 CPPE, 27 UPPE, and 8 PE caused by CTDs, were reviewed. Biomarkers and ratios showed good diagnostic performance with high area under the curve values, sensitivities, and specificities for the differential diagnosis of the multiple types of PE. According to the decision-tree analysis, the combination of adenosine deaminase (ADA), serum albumin, serum lactate dehydrogenase, total protein, PF-LDH/ADA, and PF-LDH/TP provided the best predictive capacity with an overall accuracy of 84.8%; the sensitivity and specificity for TPE diagnosis were 100% and 98.7%, respectively.

**Conclusion:**

The biomarkers and ratios showed good diagnostic performance, and a decision-tree with an overall accuracy of 84.8% was developed to differentiate the five types of PE in clinical settings.

**Supplementary Information:**

The online version contains supplementary material available at 10.1186/s12890-021-01459-w.

## Introduction

Pleural effusion (PE) is a common clinical manifestation, and about 3000 per million people in the world suffer from pleural disease [1]. The main types of PE include tuberculous PE (TPE), malignant PE (MPE), and parapneumonic effusion (PPE) [2]; besides, it is well established that connective tissue diseases (CTDs) can also cause PE [3, 4]. About one-third of tuberculosis patients showed extra-pulmonary tuberculosis (EPTB), while a quarter of them developed TPE [5]. Globally, MPE incidence is 660 per million people, resulting in more than 1 million people being affected [6]. About 57% PPE is caused due to community-acquired pneumonia (CAP) [7, 8], and approximately 1 million patients in the United States develop PPE annually [9].

Traditional microbiology and molecular biology methods (such as Xpert MTB/RIF) show poor performance when pleural fluid (PF) specimens are detected for TPE diagnosis, especially in acute setting [10, 11]. Further invasive surgery (such as pleural biopsy) can be used to detect caseating granuloma. Due to the poor preservation of tumor cells and the small sample size, the low cytological examination rate (about 60%) in the detection of MPE has become a long-term clinical problem. Thoracoscopic biopsy is a high-performance diagnostic method for both TPE and MPE, but its invasiveness limits clinical application [12]. Therefore, serum biomarkers, including adenosine deaminase (ADA), lactate dehydrogenase (LDH), C-reaction protein (CRP) and many inflammatory cytokines are used as a means of auxiliary noninvasive detection to assist clinical diagnosis. The Light’s criteria is an early standard established for the classification of exudates or transudates effusion, which involves the ratio of serum LDH (S-LDH) and PF-LDH [13]. ADA is investigated more commonly for TPE diagnosis [10, 14]. However, equivalent or higher ADA levels may occur in other types of PPE [15], thus limiting the diagnostic performance of ADA. PF-LDH and S-LDH may also be used to identify TPE and PPE [16], but their use is limited due to low sensitivity [17]. Recent studies showed that the PF-LDH/ADA and CRP/ADA ratios, interleukin (IL)-1β, IL-γ induced protein (IP)-10, interferon (IFN)-γ, IL-13, and basic fibroblast growth factor can also be used to identify TPE and PPE [16, 18, 19], while PF presepsin, CRP, and procalcitonin (PCT) levels can be used as additional tools for the assessment of the differential diagnosis of PE [18]. A combination of serum calprotectin and neutrophil gelatinase-associated lipocalin serological markers and chest X-ray constitutes a high performance assay used for differentiating CPPE from empyema [20]. To our knowledge, research has mainly focused on only a few types of PE and/or indicators, which have been extensively studied [10, 15, 17], and relatively little attention has been given to the combined application of these biomarkers and ratios for the differential diagnosis of the multiple types of PE. Although plenty of serum and PF biomarkers have been previously mentioned as potential diagnostic indicators, performance of their diagnostic value is still doubtful, especially in a clinical setting in general hospitals in China that patients with common and rare PE are included. To further understand and rationally use the potential application value of biomarkers in the diagnosis of common clinical PE, we investigated the biomarkers and their ratios in the serum and PF for the differential diagnosis of TPE, MPE, complicated PPE (CPPE), uncomplicated PPE (UPPE), and PE caused by CTDs, and developed a diagnostic strategy for PE for reference and guidance.

## Methods

### Study design

This is a retrospective survey and analysis of the biomarkers and ratios in the serum and PF for the differential diagnosis of the multiple types of PE. Inpatients, who underwent thoracentesis at Peking University People’s Hospital (PKUPH, a non-TB specialist, comprehensive teaching hospital in Beijing, China) from November 2016 to April 2019 with exudative PE according to Light’s criteria, were enrolled in this study. Exudative PE was further classified as TPE, MPE, CPPE, UPPE, or PE caused by CTDs.

### Patients

We classified exudative PE etiology into five categories. (1) TPE diagnosis was based on the presence of a caseous granuloma in the pleural biopsy and/or a positive culture for *Mycobacterium tuberculosis* (MTB) in the PF or pleural tissue with exudative PE, presenting with both clinical and radiological responses to anti-TB treatment [18]. (2) MPE was diagnosed when PF cytology or pleural biopsy was positive for malignant cells [19]. (3) PPE was defined as exudative effusion associated with bacterial pneumonia, lung abscess, or bronchiectasis, with no MTB in the PF obtained by continuous thoracentesis procedures and no evidence of MTB in the pathological manifestations of inflammatory pleuritis, pleural fibrosis and plaques, or chronic empyema [16]. PPE was further divided into UPPE, when patients responded to antibiotic treatment alone, and CPPE, when non-purulent-appearing effusions required medical interventions such as drainage [2, 16]. (4) PE caused by CTDs was defined by positive histopathology or serology with a final diagnosis of CTDs and excluding other causes of PE [4, 21–24].

### Biomarker assays

Eleven biomarkers in the serum and PF were investigated in this study. The white blood cell (WBC) count in the serum was tested using Coulter DxH800 (Beckman Coulter Inc., Miami, FL, USA); serum CRP (CRP), PF total protein (TP), PF glucose (Glu), PF ADA (ADA), PF albumin (PF-Alb), and PF-LDH levels were investigated using the routine analyzer LABOSPECT 008 (Hitachi High-Technologies, Tokyo, Japan); serum albumin (S-Alb) and S-LDH were tested using the AU5800 analyzer (Beckman Coulter Inc., Miami, FL, USA); PF total cell count was manually tested using the Neubauer counting chamber (Qijing Biochemical Instrument Co., Ltd., Shanghai, China); and PF pH was manually measured using pH paper (Sanaisi glass instrument, shanghai, China). As CRP results of some patients could not be obtained, they were excluded from CRP analysis.

### Data analysis

The Shapiro–Wilk test was used to evaluate the distribution, while the Kruskal–Wallis test was used to determine the differences in variables among the groups for non-normal distribution. A comparison between two groups was analyzed by the Mann–Whitney U test. Only parameters that yielded statistically significant *P* values < 0.01 between the two groups were entered for the construction of the receiver operating characteristic (ROC) curve. The ROC curve was analyzed, and the diagnostic accuracy was assessed from the area under the curve (AUC). Statistical analyses were performed using the SPSS software version 22.0 (SPSS Inc., Chicago, IL, USA) and MedCalc (MedCalc Software, Ostend, Belgium). Then the three parameters with the highest AUC values were enrolled in the decision-tree construction (if the number of parameters was less than 3, or the number of parallels was more than 3, all were selected). Salford Predictive Modeler 8.0 (Salford Systems, San Diego, CA, USA), a data mining platform for creating predictive models from databases and identifying the most predictive cut-off for each independent variable [25], was used to differentially diagnose these five types of PE, and cross-validation was repeated 10 times to evaluate the accuracy of the model.

## Results

### Patient characteristics

Totally 112 patients with a definitive PE diagnosis, including 25 MPE, 33 TPE, 46 PPE (19 CPPE and 27 UPPE) patients, and 8 PE caused by CTDs, were reviewed (Table 1). All information of the 11 biomarkers of 92 patients, among the 112 patients, was collected, while CRP results of 20 patients (5 TPE, 4 MPE, 3 CPPE, 7 UPPE, and 1 PE caused by CTDs) could not be obtained. The mean age of the patients was 58.4 ± 17.2 years, and 72 cases were male (64.3%).

**Table 1:** Comparison of the parameters of the five different types of pleural effusion

	TPE (n = 33)	MPE (n = 25)	PPE (n = 46)		PE caused by CTDs (n = 8)
			CPPE (n = 19)	UPPE (n = 27)	
Demographic data:					
Age, years	48 (17–86)	66 (42–90)	60 (37–79)	62 (25–88)	57.5 (44–82)
Male blood biomarkers:	22 (66.7%)	13 (52.0%)	17 (89.47%)	17 (62.96%)	3 (37.5%)
WBC, cells/μL	5500 (2700–10,400)	6700** (3800–16,000)	9790** (4400–30,700)	8260** (4000–16,100)	6385 (3600–10,100)
CRP, mg/L	37.75 (3.77–135.87)	24.22 (0.76–128.03)	108.79** (23–208.1)	41.4 (1.08–293.4)	5.14** (0.32–19.28)
S-Alb, g/dL	33.3 (24.6–40.3)	33.7 (23.1–44.3)	31.8 (24.2–39.3)	31.8 (23.7–40.8)	34.8 (23.3–40.6)
S-LDH, U/L	161 (97–605)	172 (120–302)	169 (120–860)	186 (108–990)	199.5 (144–455)
Pleural fluid biomarkers					
Total cell counts, cells/ul	4800 (416–20,000)	4120 (500–197,164)	7007 (950–290,000)	2400 (120–500,000)	1604 (100–9885)
pH	7.5 (1–8.5)	7 (5.5–8.7)	7 (6–8.5)	7.5 (6.5–9)	7.55 (6.5–8.5)
TP, g/dL	49.2 (27.4–59.6)	47.8 (30.9–60.6)	45.3 (5.1–62.3)	33.6** (15.9–58.3)	40.05 (15.5–54.1)
PF-Alb, g/dL	27.1 (18.2–34.7)	27.5 (17–34.7)	23.4* (2.7–31.5)	20** (1.9–43.7)	22* (9–25.4)
Glu, mmol/L	4.96 (0.16–11.1)	5.84 (2.67–11.03)	2.95 (0.09–11.86)	6.77** (0.25–12.78)	6.125* (5.29–12)
PF-LDH, U/L	532 (144–1783)	377 (150–2078)	1607** (731–10,613)	277** (68–1169)	136.5** (62–354)
ADA, U/L	48 (20.6–81.5)	8.9** (5.1–25.4)	32.9 (12–115.4)	8.9** (1.4–19.5)	11.2** (1.2–22.4)
Ratios between two biomarkers: only the parameters with *P* value < 0.01 between any two groups are included					
WBC/CRP	123.82 (26.5–1298.1)	307.89 (87.62–8026.32)	112.63 (53.34–363.91)	202.81 (27.88–4259.26)	1206.23 (254.15–24,062.50)
WBC/TP	113.38 (77.09–204.38)	146.63 (94.13–393.12)	217.62 (132.13–5039.22)	257.86** (88.80–763.44)	135.21 (93.26–651.61)
WBC/PF-Alb	201.33 (118.77–352.54)	255.73 (155.20–804.02)	509.32** (221.11–9518.52)	400.00** (169.12–3789.47)	304.03 (145.16–1122.22)
WBC/Glu	1138.06 (444.44–17,837.84)	1244.90 (536.91–5914.29)	3627.12 (758.85–122,280.95)	1293.63 (328.64–28,800)	861.09 (573.25–1909.26)
WBC/PF-LDH	11.02 (1.51–38.19)	20.27 (2.94–85.33)	6.15** (1.92–13.12)	37.56** (6.95–135.29)	42.43 (17.51–124.19)
WBC/ADA	119.80 (41.1–271.84)	648.94 ** (194.87–2509.80)	325.23 (140.81–744.93)	957.27** (369.23–4272.72)	473.78 (264.79–8416.67)
CRP/TP	0.83 (0.08–2.91)	0.55 (0.02–3.15)	2.44 (0.37–34.95)	1.20 (0.02–10.92)	0.17 (0.01–0.0.44)
CRP/PF-Alb	1.47 (0.15–6.97)	0.89 (0.03–6.43)	4.72** (0.73–66.02)	2.22 (0.04–55.97)	0.32 (0.01–0.79)
CRP/ADA	0.89 (0.14–3.94)	1.97 (0.08–16.06)	3.75 (0.55–10.73)	6.85 (0.09–105.04)	0.58 (0.11–2.40)
CRP/Glu	9.55 (0.69–179.08)	4.21 (0.10–45.73)	38.7 (6.54–891.44)	5.50 (0.13–425.40)	0.86** (0.03–3.42)
TP/PF-Alb	1.8 (1.01–2.39)	1.74 (1.42–2.76)	1.89 (1.63–2.54)	1.71 (0.55–8.89)	1.83 (1.36–2.63)
TP/Glu	9.61 (4.52–212.5)	7.81 (3.91–15.58)	14.96 (3.95–412.22)	5.05** (1.52–67.6)	6.31 (2.44–9.08)
TP/ADA	0.98 (0.52–2.42)	5.24 (1.60–9.37)	1.20 (0.15–3.34)	4.28** (1.2–11.36)	3.94 (2.28–12.92)
PF-Alb/Glu	5.69 (2.38–113.75)	4.27 (1.94–10.94)	6.51 (2.08–162.22)	2.59** (1.03–8.71)	3.47** (1.7–4.31)
PF-Alb/ADA	0.56 (0.28–1.29)	2.77 (0.78–5.29)	1.20 (0.15–3.34)	2.43** (0.19–8)	2.22** (0.92–7.5)
PF-LDH/TP	11.43 (2.67–52.44)	7.45 (3.14–49.95)	6.51 (2.08–162.22)	6.60 (3.21–61.3)	3.314 (1.97–9.35)
PF-LDH/CRP	15.66 (2.79–159.42)	12.88 (1.95–1109.71)	17.15 (4.48–117.26)	8.69 (0.62–198.15)	36.36 (8.09–193.75)
PF-LDH/PF-Alb	17.74 (4.43–97.97)	12.63 (5.45–80.80)	72.92** (27.17–1245.56)	10.37 (5.95–545.26)	6.07* (2.87–17.18)
PF-LDH/Glu	106.02 (21.98–11,143.75)	54.25 (18.46–778.28)	767.32 (75.89–36,596.55)	35.62 (8.69–4144.00)	23.67 (5.17–59.4)
PF-LDH/ADA	12.27 (4.48–29.34)	38.23 (8.31–183.89)	62.97 (29.87–133.92)	32.92** (12.14–101.57)	14.00 (7.43–120.83)
ADA/Glu	9.58 (2.69–410.63)	1.29 (0.70–9.07)	12.12 (1.72–397.93)	1.25** (0.21–40.8)	1.59** (0.23–3.76)

Continuous variables are presented as the median (range), and qualitative variables are presented as the number (percentage). For the ratios between two biomarkers, only the parameters with *P* value < 0.01 between any two groups are included

*TPE* tuberculous pleural effusion, *MPE* malignant pleural effusion, *CPPE* complicated parapneumonic effusion, *UPPE* uncomplicated parapneumonic effusion, *CTDs* connective tissue diseases. Blood parameters: *CRP* C-reactive protein, *S-Alb* serum albumin, *S-LDH* serum lactate dehydrogenase, *WBC* white blood cells. Pleural fluid parameters: *ADA* adenosine deaminase, *Glu* glucose, *PF-Alb* pleural fluid albumin, *PF-LDH* pleural fluid lactate dehydrogenase, *TP* total protein

*This parameter showed a significant difference between the investigated and TPE groups, *P* < 0.01

**P < 0.001

### Differentiating TPE from other causes of PE

**Fig. 1:**
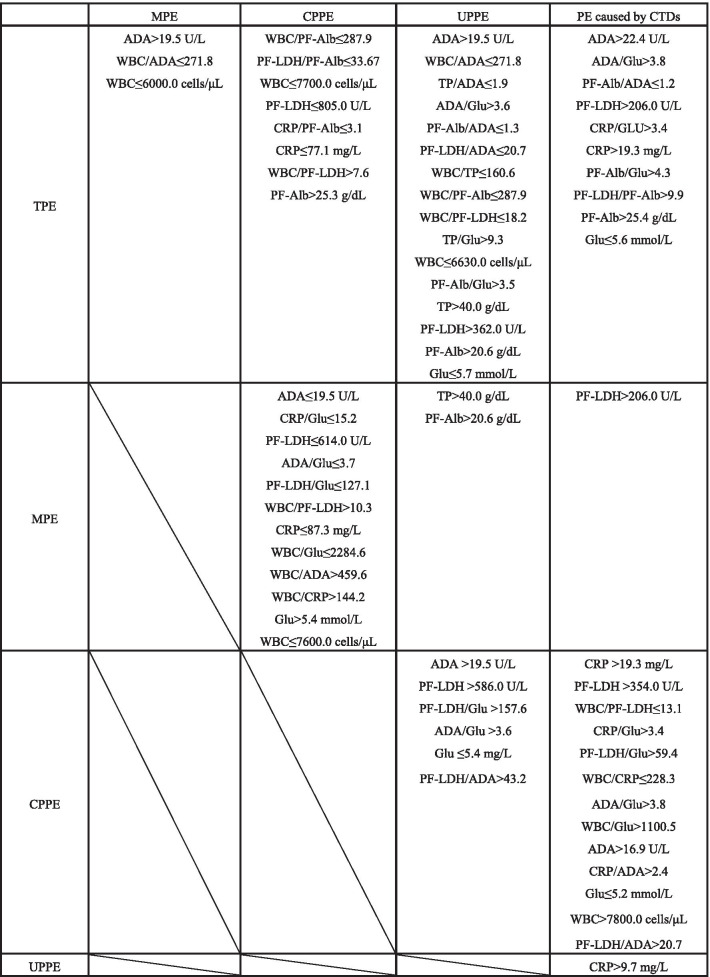
Comparison of the five different types of pleural effusion. The grid shows the biomarkers and ratios that show significant differences (*P* < 0.01) and the calculated cutoff values. These parameters are arranged in descending order of their corresponding area under the curve (AUC) values. TPE, tuberculous pleural effusion; MPE, malignant pleural effusion; CPPE, complicated parapneumonic effusion; UPPE, uncomplicated parapneumonic effusion; CTDs, connective tissue diseases. Blood parameters: CRP, C-reactive protein; S-Alb, serum albumin; S-LDH, serum lactate dehydrogenase; WBC, white blood cells. Pleural fluid parameters: ADA, adenosine deaminase; Glu, glucose; PF-Alb, pleural fluid albumin; PF-LDH, pleural fluid lactate dehydrogenase; TP, total protein

*(1) TPE versus (vs) MPE* To distinguish TPE from MPE (Table 1, Fig. 1, Additional file 1: Table S1, and Additional file 1: Fig. S1), ADA, WBC/ADA, and WBC levels were investigated, and significant differences (*P* < 0.01) were observed. According to the ROC curve, the AUC values were 0.993 for both ADA (> 19.5 U/L) and WBC/ADA (≤ 271.8). The sensitivity of both ADA and WBC/ADA was 100% (95% confidence interval [CI] was 89.4–100%), and the specificity of both was 92.0% (95% CI 74.0–99.0%). With both positive likelihood ratios (LR+) greater than 10 and negative likelihood ratios (LR-) less than 0.1 [26], both ADA (> 19.5 U/L) and WBC/ADA (≤ 271.8) can provide highly credible positive and negative results.

*(2) TPE vs CPPE* To differentiate TPE from CPPE (Table 1, Fig. 1, Additional file 1: Table S2, and Additional file 1: Fig. S2), WBC/PF-Alb (≤ 287.9) was shown to have the highest AUC value (0.968, 95% CI 0.878–0.997), with 93.9% (95% CI 79.8–99.3%) sensitivity and 89.5% (95% CI 66.9–98.7%) specificity. PF-LDH/PF-Alb (≤ 33.67) and WBC (≤ 7700.0 cells/μL) also showed high AUC values (> 0.94), with high sensitivities (> 90%) and specificities (> 94%).

*(3) TPE vs UPPE* To differentiate TPE from UPPE (Table 1, Fig. 1, Additional file 1: Table S3, and Additional file 1: Fig. S3), ADA (> 19.5 U/L) and WBC/ADA (≤ 271.8) were shown to have the largest AUC value (1), with 100% (95% CI 89.4–100%) sensitivity and 100% (95% CI 87.2–100%) specificity. TP/ADA(≤ 1.9) and ADA/Glu (> 3.6) also showed high AUC values (> 0.96), with high sensitivities (> 93%) and specificities (> 92%).

*(4) TPE vs PE caused by CTDs* To differentiate TPE from PE caused by CTDs (Table 1, Fig. 1, Additional file 1: Table S4, and Additional file 1: Fig. S4), ADA (> 22.4 U/L) and ADA/Glu (> 3.8) were shown to have the highest AUC value (0.989), with high sensitivities (> 90%) and 100% specificities. PF-Alb/ADA (≤ 1.2) and PF-LDH (> 206.0 U/L) also showed high AUC values (> 0.95), with 93.9% sensitivity and 87.5% specificity.

### Differential diagnosis of MPE, CPPE, UPPE, and PE caused by CTDs

*(5) MPE vs CPPE* When the MPE and CPPE groups were compared (Table 1, Fig. 1, Additional file 1: Table S5, and Additional file 1: Fig. S5), ADA (≤ 19.5 U/L) showed the best diagnostic performance (AUC = 0.955), with 92.0% (95% CI 74.0–99.0%) sensitivity and 89.5% (95% CI 66.9–98.7%) specificity. CRP/Glu (≤ 15.2), PF-LDH (≤ 614.0 U/L), ADA/Glu (≤ 3.7), PF-LDH/Glu (≤ 127.1), and WBC/PF-LDH (> 10.3) also showed high AUC values (> 0.91).

*(6) MPE vs UPPE* To differentiate MPE from UPPE (Table 1, Fig. 1, Additional file 1: Table S6, and Additional file 1: Fig. S6), TP (> 40.0 g/dL) was shown to have the largest AUC value (0.788), with 88.0% (95% CI 68.8–97.5%) sensitivity and 70.4% (95% CI 49.8–86.2%) specificity. PF-Alb (> 20.6 g/dL) (AUC = 0.750) showed 84.0% (95% CI 63.9–95.5%) sensitivity and 59.3% (95% CI 38.8–77.6%) specificity.

*(7) MPE vs PE caused by CTDs* Statistically significant difference in only the PF-LDH levels between the PE caused by CTDs and MPE groups was observed (AUC = 0.895) (*P* < 0.01) (Table 1, Fig. 1, Additional file 1: Table S7, and Additional file 1: Fig. S7). At a cutoff value of > 206.0 U/L, PF-LDH showed 76.0% (95% CI 54.9–90.6%) sensitivity and 87.5% (95% CI 47.3–99.7%) specificity.

*(8) CPPE vs UPPE *On comparing the CPPE and UPPE groups (Table 1, Fig. 1, Additional file 1: Table S8, and Additional file 1: Fig. S8), ADA (> 19.5 U/L), PF-LDH (> 586.0 U/L), PF-LDH/Glu (> 157.6), and ADA/Glu (> 3.6) were all found to show high AUC values (> 0.94), with high sensitivities (> 84%) and specificities (> 88%).

*(9) CPPE vs PE caused by CTDs* To distinguish CPPE from PE caused by CTDs (Table 1, Fig. 1, Additional file 1: Table S9, and Additional file 1: Fig. S9), CRP (> 19.3 mg/L), PF-LDH (> 354.0 U/L), WBC/PF-LDH (≤ 13.1), CRP/Glu (> 3.4), and PF-LDH/Glu (> 59.4) were all shown to have the best diagnostic performances (AUC = 1), with 100% sensitivity and 100% specificity.

*(10) UPPE vs PE caused by CTDs* Significant difference in only the CRP levels between the PE caused by CTDs and UPPE groups was observed (AUC = 0.871) (*P* < 0.01) (Table 1, Fig. 1, Additional file 1: Table S10, and Additional file 1: Fig. S10). At a cutoff value of > 9.74 mg/L, CRP showed 85.0% (95% CI 62.1–96.8%) sensitivity and 85.7% (95% CI 42.1–99.6%) specificity.

### Differential diagnosis of the five types of PE by the decision-tree analysis

**Fig. 2:**
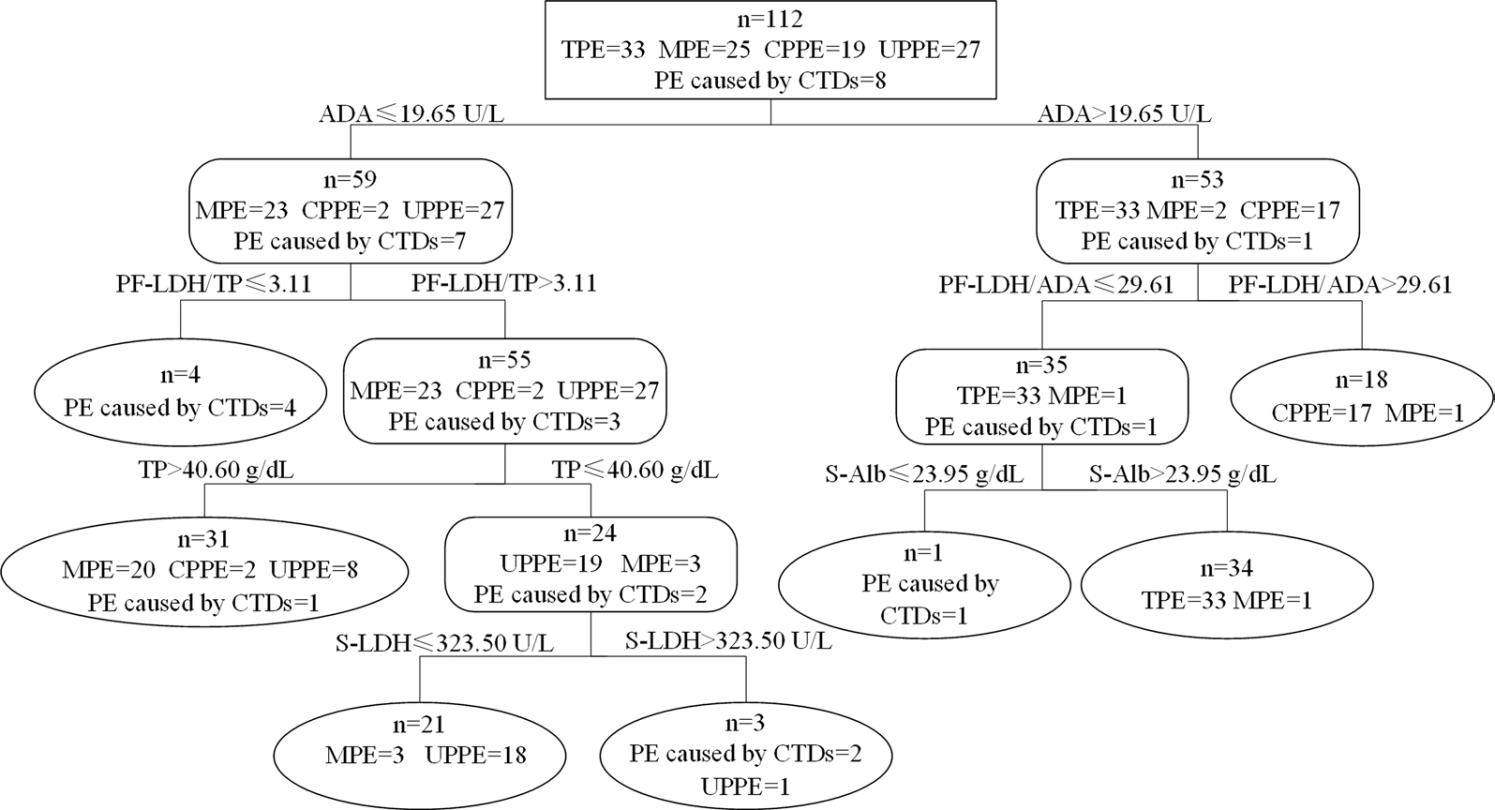
Differential diagnosis of the five types of pleural effusion by decision-tree analysis. The combination of ADA, S-Alb, S-LDH, TP, PF-LDH/ADA, and PF-LDH/TP provided the best predictive capacity, with an overall accuracy of 84.8% (95/112). TPE, tuberculous pleural effusion; MPE, malignant pleural effusion; CPPE, complicated parapneumonic effusion; UPPE, uncomplicated parapneumonic effusion; CTDs, connective tissue diseases. ADA, pleural fluid adenosine deaminase; PF-LDH, pleural fluid lactate dehydrogenase; S-Alb, serum albumin; S-LDH, serum lactate dehydrogenase; TP, pleural fluid total protein

The biomarkers and ratios were subjected to the decision-tree analysis, and the combination of ADA, S-Alb, S-LDH, TP, PF-LDH/ADA, and PF-LDH/TP were found to provide the best predictive capacity (Fig. 2). At cutoff values of ADA > 19.65 U/L, PF-LDH/ADA ≤ 29.61, and S-Alb > 23.95 g/dL, the sensitivity, specificity, positive predictive value (PPV), and negative predictive value (NPV) for TPE diagnosis were 100% (95% CI 89.6–100%), 98.7% (95% CI 93.2–100%), 97.1% (95% CI 85.1–99.6%), and 100.% (95% CI 95.3–100%), respectively (Table 2). Similarly, the sensitivity, specificity, PPV, and NPV for MPE diagnosis were 80.0%, 87.4%, 64.5%, and 93.8%, respectively; for CPPE diagnosis were 89.5%, 98.9%, 94.4%, and 97.9%, respectively; for UPPE diagnosis were 66.7%, 96.5%, 85.7%, and 90.1%, respectively; and for the diagnosis of PE caused by CTDs were 87.5%, 99.0%, 87.5%, and 99.0%, respectively. The overall accuracy of the decision-tree analysis was 84.8% (95/112, 95% CI 77.0–90.3%).

**Table 2:** The sensitivity, specificity, positive predictive values, and negative predictive values of the decision-tree analysis to distinguish five types of pleural effusion

Target	Sensitivity (%) (95% CI)	Specificity (%) (95% CI)	PPV (95% CI)	NPV (95% CI)
TPE	100.0 (89.6–100.0)	98.7 (93.2–100.0)	97.1 (85.1–99.6)	100.0 (95.3–100.0)
MPE	80.0 (60.9–91.1)	87.4 (78.8–92.8)	64.5 (47.0–78.9)	93.8 (86.4–97.3)
CPPE	89.5 (68.6–97.1)	98.9 (94.2–99.8)	94.4 (74.2–99.0)	97.9 (92.6–99.4)
UPPE	66.7 (47.8–81.4)	96.5 (90.1–98.8)	85.7 (65.4–95.0)	90.1 (82.3–94.7)
PE caused by CTDs	87.5 (52.9–97.8)	99.0 (94.8–99.8)	87.5 (52.9–97.8)	99.0 (94.8–99.8)

*TPE* tuberculous pleural effusion, *MPE* malignant pleural effusion, *CPPE* complicated parapneumonic effusion, *UPPE* uncomplicated parapneumonic effusion, *CTDs* connective tissue diseases, *CI* confidence interval, *PPV* positive predictive value, *NPV* negative predictive value

## Discussion

ADA showed good diagnostic performance (AUC > 0.98) in differentiating TPE from MPE, UPPE, and PE caused by CTDs, and this finding supported evidences from previous studies [10, 16, 19, 27]. It is now well established that the observed increase in ADA, attributed to the rise in the levels of different types of ADA: increased ADA levels in tuberculosis, is primarily due to an increase in the activity of ADA isoenzyme (ADA-2), which is only found in monocytes-macrophages. However, high levels of ADA-1 are usually associated with CPPE or empyema [16, 28, 29]. Beside its application in tuberculosis diagnosis, ADA also showed great diagnostic value in distinguishing MPE and CPPE, CPPE and UPPE, and CPPE and PE caused by CTDs (AUC > 0.93), as mentioned previously [16, 30]. The diagnostic values of ADA were further examined by using likelihood ratios (LR). Agreed with those evaluated by ROC curves, ADA showed both high LR+ (> 10) and low LR− (< 0.1) in distinguish TPE from MPE, UPPE, and PE caused by CTDs (Table S1, S3 and S4). However, it is still difficult to distinguish TPE from CPPE based on ADA alone.

A significant association of high PF-LDH level with high degree of necrosis in pleural cavity has been observed previously [31]. In our study, PF-LDH played an important role in differentiating CPPE and TPE from the other three types of PE, thus expanding previous research [10, 30, 32]. Significant difference in only the PF-LDH levels between the PE caused by CTDs and MPE groups (*P* < 0.001) was observed, and this might be due to the LDH released from cells that were invaded and destroyed by the tumor; meanwhile, tumor cells preferentially use glycolysis, rather than oxidative phosphorylation (a switch, which is mediated by LDH, in the ATP generation pathway), to obtain energy [31, 33–36]. In future studies, we must investigate more immunological and oncological indicators for MPE diagnosis by taking clinical diagnosis into account. Previous study believed that the use of biomarkers in MPE diagnosis is still limited due to inadequate validation [37]. However, this study revealed promising biomarkers in the diagnosis of MPE, including PF-LDH, ADA, TP, and so on, thereby strengthening the use of biomarkers in MPE diagnosis and promoted a further understanding of the clinical application of biomarkers.

As a widely used diagnostic indicator for differentiating infectious and non-infectious diseases, (serum) CRP showed high clinical diagnostic value (AUC > 0.87) in differentiating infectious PE (TPE, CPPE, and UPPE) from PE caused by CTDs. This result extended the findings of previous investigations, which reported the value of PF-CRP in diagnosing infectious effusions (AUC = 0.82) [18], and a high AUC (0.899) also observed in (serum) CRP for differentiating MPE and CPPE [38]. Significant difference in only CRP levels (AUC = 0.871) between the PE caused by CTDs and UPPE groups was observed; thus, the clinical diagnostic, traditional microbiology culture method and immunological biomarkers remain essential. Interestingly, CRP, CRP/PF-Alb, CRP/Glu, CRP/ADA, and WBC/CRP showed great value in distinguishing CPPE from TPE, MPE, and PE caused by CTDs, which seems to imply CRP-based CPPE identification strategies could be developed. However, the CRP value is affected by many factors, for instance, inflammation caused by injury, infection, and autoimmune diseases can lead to increased (serum) CRP levels [39–42]; other factors, including smoking and obesity, can also lead to high levels of CRP [43, 44]. Therefore, the universality of using serum CRP for PE identification is limited, and its clinical application should be cautious.

According to the diagnostic classification tree, the combination of ADA, S-Alb, S-LDH, TP, PF-LDH/ADA, and PF-LDH/TP provided the best predictive capacity, with an overall accuracy of 84.8%, thus showing great potential in the clinical differential diagnosis of the five types of PE, especially TPE (100% sensitivity and 98.7% specificity).

Considering the poor performance of the traditional tuberculosis culture (time-consuming) and molecular techniques (including Xpert MTB/RIF, showed low sensitivity) in detecting TPE, the method of integrated biomarkers and ratios provided a strategy for rapid and accurate TPE diagnosis, and could be clinically practiced. Little was known about the biomarkers of PE caused by CTDs previously, and this study suggested that the combination of a few biomarkers and ratios could provide a diagnostic strategy for PE caused by CTDs with 87.5% sensitivity and 99.0% specificity. Moreover, UPPE (29.6%, 8/27), CPPE (10.5%, 2/19), and PE caused by CTDs (12.5%, 1/8) could be misdiagnosed as MPE, while MPE could be misdiagnosed as UPPE (12.0%, 3/25), TPE (4.0%, 1/25), or CPPE (4.0%, 1/25), implying that there were more concerns in the clinical differential diagnosis of MPE. The sensitivity for UPPE diagnosis was only 66.6%; thus, microbial culture was still found to be necessary to detect UPPE. The total accuracy was only slightly lower in this study than in previous studies (94.6%-96.6%), which only reported the differentiation of TPE from MPE by investigating QuantiFERON-TB Gold In-Tube (QFT-GIT), pleural ADA, PF-LDH, and a few demographic factors (age, fever) [45, 46]. However, for patients with unknown PE in clinical settings, the decision-tree analysis developed in this study could help in diagnosing more types of PE and provide doctors with a more detailed diagnostic guidance.

Since there are few studies that use biomarkers (and their ratios) and decision-tree to assist in the diagnosis of multiple types of PE in China, this research could help clinicians better perform early diagnosis and treatment (especially for TPE, due to the limitation of traditional methods, and for CPPE, as the patients requiring surgery), reduce invasive medical operations, and provide a reference for the research of using biomarkers to assist in the diagnosis of PE in general hospitals in China. It also provides a foundation for future multi-center, large-scale and in-depth research.

### Study limitations

This study has some limitations. First, it was a retrospective study performed in a single center. Second, the study included a relatively small number of patients with PE caused by CTDs (n = 8), thus limited the relevant scope of our findings. For instance, PF-LDH showed a high diagnostic performance in differentiating MPE and PE caused by CTDs; however, whether it is suitable to be used as a clinical diagnostic index for other CTD patients must be verified. Third, the absence of CRP results of a few patients (n = 20) produced uncertain results based on the CRP level and corresponding ratio. However, since the sensitivity and specificity of the cutoff values and corresponding 95% CI between pairs of the different types of PE groups were obtained, future prospective studies that cover a larger sample size and more comprehensive parameters can be designed.

## Conclusions

In this study, we investigated the significant differences in the biomarkers and ratios, such as ADA, CRP, PF-Alb, PF-LDH, WBC, WBC/PF-LDH, and PF-LDH/ADA, between pairs of the different types of PE groups and developed a decision-tree with an overall accuracy of 84.8% to help differentially diagnose the five types of PE in clinical settings. Notably, the strategy with cutoff values of ADA > 19.65 U/L, PF-LDH/ADA ≤ 29.61, and S-Alb > 23.95 g/dL provided 100% sensitivity and 98.7% specificity for the differential diagnosis of TPE. Decision-tree analysis is a comprehensive and rapid method that based on serum/PF biomarkers/ratios routinely assessed in clinical practice, which could provide more help in early target treatment and appropriate patient care, contributing to better prevention of disease progression.

## Supplementary Information

**Additional file 1: **Supplementary figures and tables mentioned in the main text (Figures S1–S10 and Tables S1–S10). 

## Data Availability

The full data and materials can be obtained from Dr. Li (Shuguang Li) upon sufficient and reasonable request.
