# Supplementary material for: A retrospective study on the combined biomarkers and ratios in serum and pleural fluid to distinguish the multiple types of pleural effusion
Source: BMC Pulm Med. 2021 Mar 19;21:95. doi: 10.1186/s12890-021-01459-w (PMC7980630; doi:10.1186/s12890-021-01459-w)
Supplement: Supplementary file 1 — Additional file 1: Supplementary figures and tables mentioned in the main text (Figures S1–S10 and Tables S1–S10). [file 12890_2021_1459_MOESM1_ESM.doc]

**SUPPLEMENTARY INFORMATION**

**A retrospective study on the combined biomarkers and ratios in serum and pleural fluid to distinguish the multiple types of pleural effusion**

Liyan Lin^a,b,$^, Shuguang Li^a,$,*^, Qiao Xiong^c^, and Hui Wang^a,*^

^a^Department of Clinical Laboratory, Peking University People’s Hospital, Beijing, 100044, China

^b^Department of Infectious Diseases and Immunology, Sydney Medical School, The University of Sydney, Sydney, 2006, Australia

^c^School of Public Health, The University of Sydney, Sydney, 2006, Australia

^$^Liyan Lin and Shuguang Li contributed equally to this work.

^*^Correspondence: Shuguang Li, Email: [sdu_lsg@126.com](mailto:sdu_lsg@126.com);

Hui Wang, Email: [wanghui@pkuph.edu.cn](mailto:wanghui@pkuph.edu.cn) and [whuibj@163.com](mailto:whuibj@163.com).

Address: Peking University People’s Hospital, Xizhimen South Avenue No. 11, Beijing, 100044, China.

Tel: +86-010-88326300, +86-010-88326306

**Figure S1***- Receiver operating characteristic (ROC) curve* *for the biomarkers and their ratios to differentiate tuberculous pleural effusion (TPE) and malignant pleural effusion (MPE).*


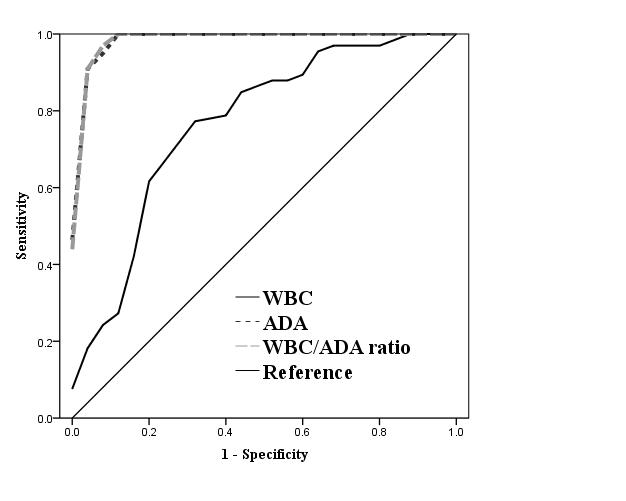


Figure legend: ROC curves for the biomarkers and their ratios to differentiate TPE and MPE groups. Only parameters that yielded statistically significant *P*-values <0.01 between the two groups were entered for the construction of the ROC curve.

**Figure S2***- Receiver operating characteristic (ROC) curve* *for the biomarkers and their ratios to differentiate tuberculous pleural effusion (TPE) and complicated parapneumonic effusion (CPPE).*

*
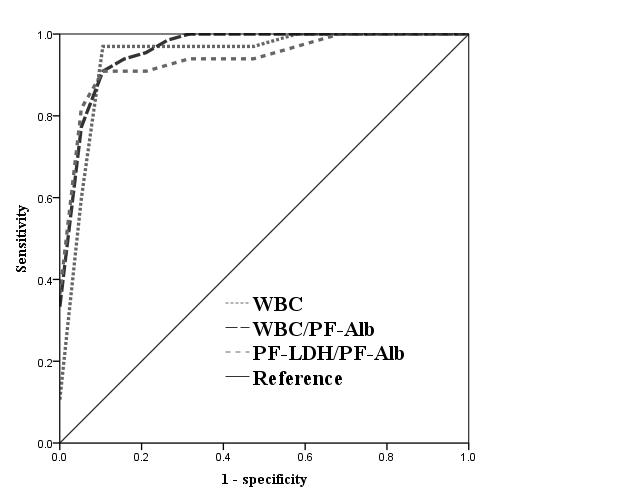
*

Figure legend: ROC curves for the biomarkers and their ratios to differentiate TPE and CPPE groups. Only parameters that yielded statistically significant *P*-values <0.01 between the two groups were entered for the construction of the ROC curve. For simplicity and clarity, only the three parameters with the highest AUC values were shown.

**Figure S3***- Receiver operating characteristic (ROC) curve* *for the biomarkers and their ratios to differentiate tuberculous pleural effusion (TPE) and uncomplicated parapneumonic effusion (UPPE).*

*
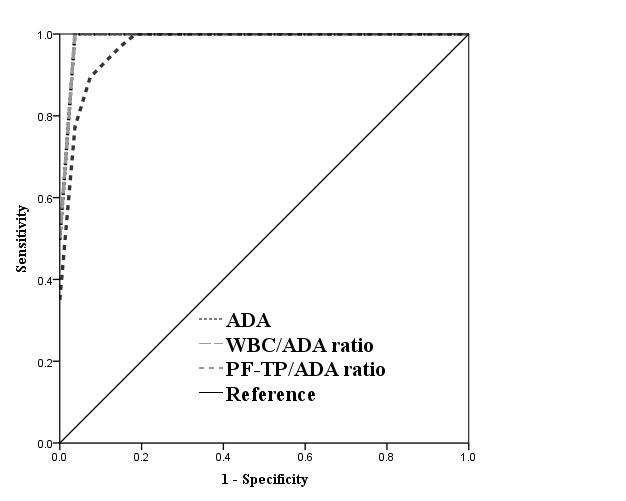
*

Figure legend: ROC curves for the biomarkers and their ratios to differentiate TPE and UPPE groups. Only parameters that yielded statistically significant *P*-values <0.01 between the two groups were entered for the construction of the ROC curve. For simplicity and clarity, only the three parameters with the highest AUC values were shown.

**Figure S4***- Receiver operating characteristic (ROC) curve* *for the biomarkers and their ratios to differentiate tuberculous pleural effusion (TPE) and PE caused by connective tissue diseases (CTDs).*

*
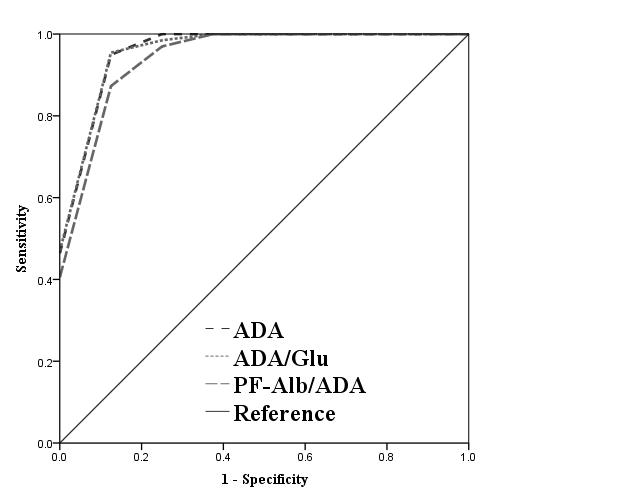
*

Figure legend: ROC curves for the biomarkers and their ratios to differentiate TPE and PE caused by CTDs. Only parameters that yielded statistically significant *P*-values <0.01 between the two groups were entered for the construction of the ROC curve. For simplicity and clarity, only the three parameters with the highest AUC values were shown.

**Figure S5***- Receiver operating characteristic (ROC) curve* *for the biomarkers and their ratios to differentiate malignant pleural effusion (MPE) and complicated parapneumonic effusion (CPPE).
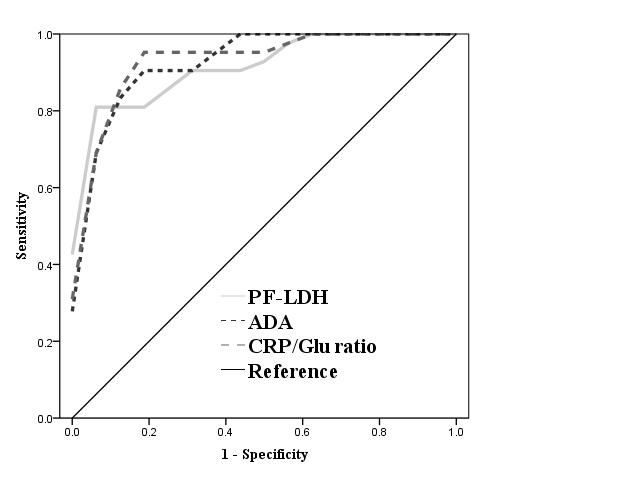
*

Figure legend: ROC curves for the biomarkers and their ratios to differentiate MPE and CPPE groups. Only parameters that yielded statistically significant *P*-values <0.01 between the two groups were entered for the construction of the ROC curve. For simplicity and clarity, only the three parameters with the highest AUC values were shown.

**Figure S6***- Receiver operating characteristic (ROC) curve* *for the biomarkers and their ratios to differentiate malignant pleural effusion (MPE) and uncomplicated parapneumonic effusion (UPPE).*

*
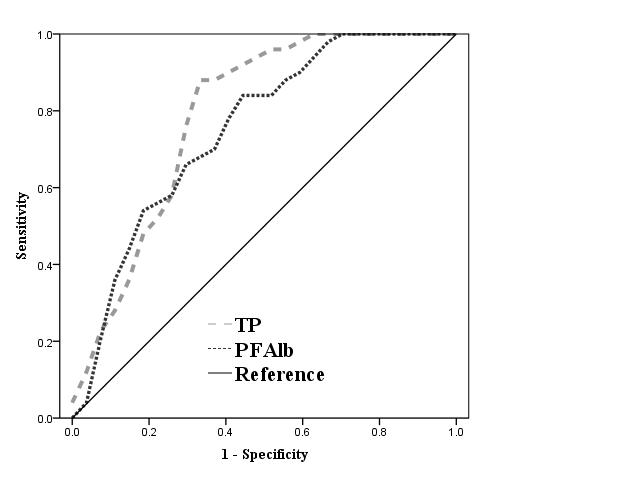
*

Figure legend: ROC curves for the biomarkers and their ratios to differentiate MPE and UPPE groups. Only parameters that yielded statistically significant *P*-values <0.01 between the two groups were entered for the construction of the ROC curve.

**Figure S7***- Receiver operating characteristic (ROC) curve* *for the biomarkers and their ratios to differentiate malignant pleural effusion (MPE) and PE caused by connective tissue diseases (CTDs).*

*
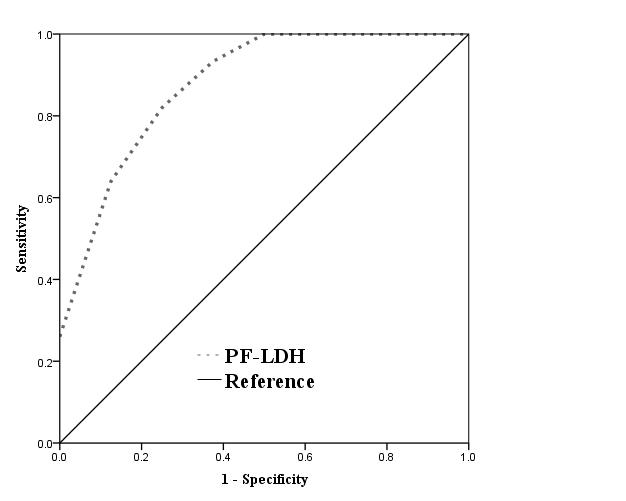
*

Figure legend: ROC curves for the biomarkers and their ratios to differentiate MPE and PE caused by CTDs. Only parameters that yielded statistically significant *P*-values <0.01 between the two groups were entered for the construction of the ROC curve.

**Figure S8***- Receiver operating characteristic (ROC) curve* *for the biomarkers and their ratios to differentiate complicated parapneumonic effusion (CPPE) and uncomplicated parapneumonic effusion (UPPE).*

*
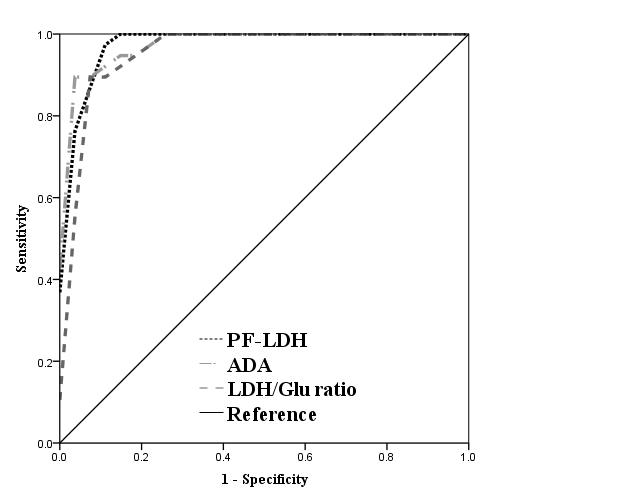
*

Figure legend: ROC curves for the biomarkers and their ratios to differentiate CPPE and UPPE groups. Only parameters that yielded statistically significant *P*-values <0.01 between the two groups were entered for the construction of the ROC curve. For simplicity and clarity, only the three parameters with the highest AUC values were shown.

**Figure S9***- Receiver operating characteristic (ROC) curve* *for the biomarkers and their ratios to differentiate complicated parapneumonic effusion (CPPE) and PE caused by connective tissue diseases (CTDs).*

*
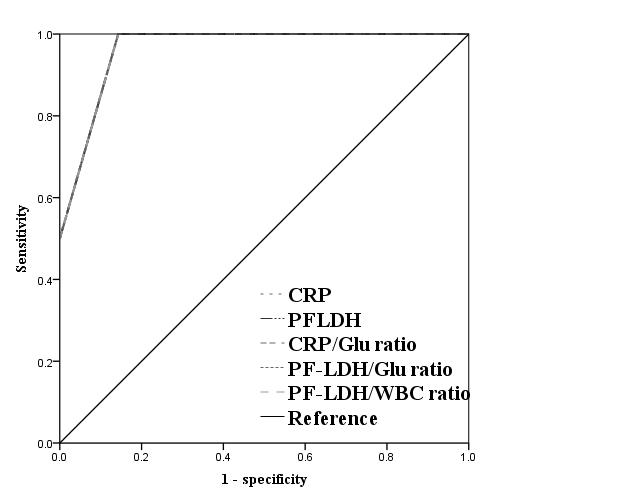
*

Figure legend: ROC curves for the biomarkers and their ratios to differentiate CPPE and PE caused by CTDs. Only parameters that yielded statistically significant *P*-values <0.01 between the two groups were entered for the construction of the ROC curve. For simplicity and clarity, only the five parameters with the highest AUC values were shown.

**Figure S10***- Receiver operating characteristic (ROC) curve* *for the biomarkers and their ratios to differentiate uncomplicated parapneumonic effusion (UPPE) and PE caused by connective tissue diseases (CTDs).*

*
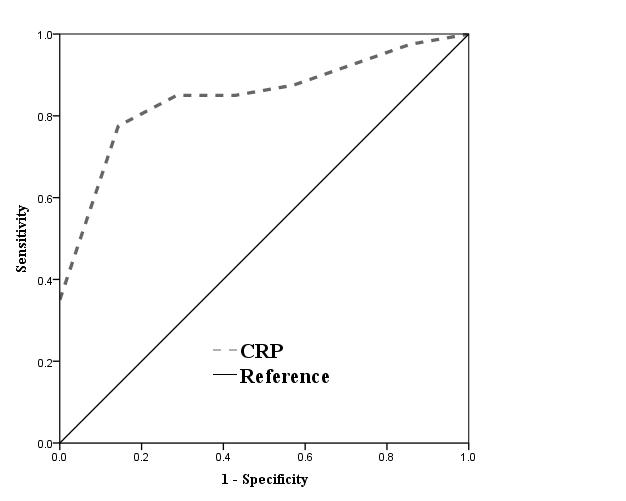
*

Figure legend: ROC curves for the biomarkers and their ratios to differentiate UPPE and PE caused by CTDs. Only parameters that yielded statistically significant *P*-values <0.01 between the two groups were entered for the construction of the ROC curve.

**Table S1-** *Parameters that yielded statistically significant difference between tuberculous pleural effusion (TPE) and malignant pleural effusion (MPE).*

| Parameters | Cutoff | Sensitivity (%)  (95 % CI) | Specificity (%)  (95 % CI) | LR+ (95% CI) | LR- (95% CI) | AUC (95% CI) |
| --- | --- | --- | --- | --- | --- | --- |
| Blood value: | | | | | | |
| WBC, cells/μL | ≤6000.0 | 69.7 (51.3 - 84.4) | 80.0 (59.3 - 93.2) | 3.48 (2.6 - 4.7) | 0.38 (0.1 - 1.0) | 0.772 (0.642-0.872) |
| Pleural fluid value: | | | | | | |
| ADA, U/L | >19.5 | 100 (89.4 - 100.0) | 92.0 (74.0 - 99.0) | 12.5 (11.1 - 14.0) | 0 | 0.993 (0.925-1) |
| Ratio: |  |  |  |  |  |  |
| WBC/ADA | ≤271.8 | 100 (89.4 - 100.0) | 92.0 (74.0 - 99.0) | 12.5 (11.1 - 14.0) | 0 | 0.993 (0.925-1) |

Table legend: The biomarkers and their ratios were compared between TPE and MPE groups. Only parameters that yielded statistically significant *P*-values <0.01 between the two groups were shown, and entered for the construction of the receiver operating characteristic (ROC) curve. The ROC curve was analyzed, and the diagnostic accuracy of the calculated cutoff value was assessed from the area under the curve (AUC) value. CI, confidence interval; LR, likelihood ratio.

**Table S2-** *Parameters that yielded statistically significant difference between tuberculous pleural effusion (TPE) and complicated parapneumonic effusion (CPPE).*

| Parameters | Cutoff | Sensitivity (%)  (95 % CI) | Specificity (%)  (95 % CI) | LR+ (95% CI) | LR- (95% CI) | AUC (95% CI) |
| --- | --- | --- | --- | --- | --- | --- |
| Blood values: | | | | | | |
| WBC, cells/μL | ≤7700.0 | 97.0 (84.2 - 99.9) | 94.7 (74.0 - 99.9) | 18.42 (16.3 - 20.8) | 0.032 (0.002 - 0.5) | 0.944 (0.843-0.989) |
| CRP, mg/L | ≤77.1 | 75.0 (55.1 - 89.3) | 87.5 (61.7 - 98.4) | 6.0 (4.5 - 8.0) | 0.29 (0.07 - 1.2) | 0.842 (0.7-0.934) |
| Pleural fluid values: | | | | | | |
| PF-Alb, g/dL | >25.3 | 72.7 (54.5 - 86.7) | 79.0 (54.4 - 93.9) | 3.45 (2.5 - 4.7) | 0.35 (0.1 - 1.0) | 0.750 (0.611-0.86) |
| PF-LDH, U/L | ≤805.0 | 78.8 (61.1 - 91.0) | 94.7 (74.0 - 99.9) | 14.97 (12.2 - 18.4) | 0.22 (0.03 - 1.7) | 0.931 (0.826-0.983) |
| Ratios: | | | | | | |
| WBC/PF-Alb | ≤287.9 | 93.9 (79.8 - 99.3) | 89.5 (66.9 - 98.7) | 8.92 (7.5 - 10.7) | 0.068 (0.01 - 0.4) | 0.968 (0.878-0.997) |
| WBC/PF-LDH | >7.6 | 72.7 (54.5 - 86.7) | 73.8 (48.8 - 90.9) | 2.76 (2.0 - 3.9) | 0.37 (0.1 - 0.9) | 0.770 (0.633-0.876) |
| CRP/PF-Alb | ≤3.1 | 78.6 (59.0 - 91.7) | 87.5 (61.7 - 98.4) | 6.29 (4.8 - 8.2) | 0.24 (0.06 - 1.1) | 0.866 (0.729-0.95) |
| PF-LDH/PF-Alb | ≤33.7 | 90.9 (75.7 – 98.1) | 94.7 (74.0 – 99.9) | 17.27 (14.8 – 20.1) | 0.096 (0.01 - 0.9) | 0.947 (0.848-0.99) |

Table legend: The biomarkers and their ratios were compared between TPE and CPPE groups. Only parameters that yielded statistically significant *P*-values <0.01 between the two groups were shown, and entered for the construction of the receiver operating characteristic (ROC) curve. The ROC curve was analyzed, and the diagnostic accuracy of the calculated cutoff value was assessed from the area under the curve (AUC) value. CI, confidence interval; LR, likelihood ratio.

**Table S3-** *Parameters that yielded statistically significant difference between tuberculous pleural effusion (TPE) and uncomplicated parapneumonic effusion (UPPE).*

| Parameters | Cutoff | Sensitivity (%)  (95 % CI) | Specificity (%)  (95 % CI) | LR+ (95% CI) | LR- (95% CI) | AUC (95% CI) |
| --- | --- | --- | --- | --- | --- | --- |
| Blood value: | | | | | | |
| WBC, cells/μL | ≤6630.0 | 87.9 (71.8 - 96.6) | 74.1 (53.7 - 88.9) | 3.39 (2.6 - 4.4) | 0.16 (0.05 - 0.5) | 0.816 (0.695-0.904) |
| Pleural fluid values: | | | | | | |
| TP, g/dL | >40.0 | 87.9 (71.8 - 96.6) | 70.4 (49.8 - 86.2) | 2.97 (2.3 - 3.9) | 0.17 (0.06 - 0.5) | 0.802 (0.679-0.894) |
| PF-Alb, g/dL | >20.6 | 90.9 (75.7 – 98.1) | 59.3 (38.8 - 77.6) | 2.23 (1.6 - 3.1) | 0.15 (0.05 - 0.5) | 0.773 (0.646-0.871) |
| Glu, mmol/L | ≤5.7 | 81.8 (64.5 - 93.0) | 70.4 (49.8 - 86.2) | 2.76 (2.1 - 3.7) | 0.26 (0.1 - 0.7) | 0.773 (0.646-0.871) |
| PF-LDH, U/L | >362.0 | 72.7 (54.5 - 86.7) | 70.4 (49.8 - 86.2) | 2.45 (1.8 - 3.4) | 0.39 (0.2 - 0.9) | 0.745 (0.616-0.849) |
| ADA, U/L | >19.5 | 100 (89.4 - 100.0) | 100 (87.2 - 100.0) | NA | 0 | 1 (0.94-1) |
| Ratios: | | | | | | |
| WBC/TP | ≤160.6 | 93.9 (79.8 - 99.3) | 77.8 (57.7 - 91.4) | 4.23 (3.4 - 5.3) | 0.078 (0.02 - 0.4) | 0.912 (0.811-0.97) |
| WBC/PF-Alb | ≤287.9 | 93.9 (79.8 – 99.3) | 77.8 (57.7 - 91.4) | 4.23 (3.4 - 5.3) | 0.078 (0.02 - 0.4) | 0.895 (0.788-0.959) |
| WBC/PF-LDH | ≤18.2 | 84.9 (68.1 - 94.9) | 81.5 (61.9 - 93.7) | 4.58 (3.6 - 5.8) | 0.19 (0.06 - 0.6) | 0.862 (0.748-0.937) |
| WBC/ADA | ≤271.8 | 100 (89.4 - 100.0) | 100 (87.2 - 100.0) | NA | 0 | 1 (0.94-1) |
| TP/Glu | >9.3 | 66.7 (48.2 - 82.0) | 92.6 (75.7 - 99.1) | 9 (6.9 - 11.7) | 0.36 (0.09 - 1.5) | 0.848 (0.732-0.928) |
| TP/ADA | ≤1.9 | 93.9 (79.8 - 99.3) | 92.6 (75.7 - 99.1) | 12.68 (11.1 - 14.6) | 0.065 (0.010 - 0.4) | 0.979 (0.903-0.999) |
| PF-Alb/Glu | >3.5 | 87.9 (71.8 - 96.6) | 63.0 (42.4 - 80.6) | 2.37 (1.7 - 3.3) | 0.19 (0.07 - 0.5) | 0.810 (0.688-0.9) |
| PF-Alb/ADA | ≤1.3 | 100 (89.4 - 100.0) | 85.2 (66.3 - 95.8) | 6.75 (5.8 - 7.9) | 0 | 0.947 (0.857-0.988) |
| PF-LDH/ADA | ≤20.7 | 90.9 (75.7 - 98.1) | 85.2 (66.3 - 95.8) | 6.14 (5.1 - 7.4) | 0.11 (0.03 - 0.4) | 0.935 (0.84-0.982) |
| ADA/Glu | >3.6 | 97.0 (84.2 - 99.9) | 92.6 (75.7 - 99.1) | 13.09 (11.6 - 14.8) | 0.033 (0.003 - 0.3) | 0.962 (0.878-0.994) |

Table legend: The biomarkers and their ratios were compared between TPE and UPPE groups. Only parameters that yielded statistically significant *P*-values <0.01 between the two groups were shown, and entered for the construction of the receiver operating characteristic (ROC) curve. The ROC curve was analyzed, and the diagnostic accuracy of the calculated cutoff value was assessed from the area under the curve (AUC) value. CI, confidence interval; LR, likelihood ratio.

**Table S4-** *Parameters that yielded statistically significant difference between tuberculous pleural effusion (TPE) and PE caused by connective tissue diseases (CTDs).*

| Parameters | Cutoff | Sensitivity (%)  (95 % CI) | Specificity (%)  (95 % CI) | LR+ (95% CI) | LR- (95% CI) | AUC (95% CI) |
| --- | --- | --- | --- | --- | --- | --- |
| Blood value: | | | | | | |
| CRP, mg/L | >19.3 | 75.0 (55.1-89.3) | 100 (59.0 - 100.0) | NA | 0.25 | 0.908 (0.761-0.979) |
| Pleural fluid values: | | | | | | |
| PF-Alb, g/dL | >25.4 | 72.7 (54.5 – 86.7) | 100 (63.1 - 100) | NA | 0.27 | 0.845 (0.698-0.939) |
| Glu, mmol/L | ≤5.6 | 75.8 (57.7 - 88.9) | 87.5 (47.3 - 99.7) | 6.06 (4.4 - 8.4) | 0.28 (0.04 - 1.9) | 0.837 (0.689-0.934) |
| PF-LDH, U/L | >206.0 | 93.9 (79.8 - 99.3) | 87.5 (47.3 - 99.7) | 7.52 (5.7 - 9.9) | 0.069 (0.007 - 0.7) | 0.955 (0.839-0.995) |
| ADA, U/L | >22.4 | 90.9 (75.7 - 98.1) | 100 (63.1 - 100.0) | NA | 0.091 | 0.989 (0.893-1) |
| Ratios: | | | | | | |
| CRP/GLU | >3.4 | 75.0 (55.1 - 89.3) | 100 (59.0 - 100.0) | NA | 0.25 | 0.918 (0.775-0.984) |
| PF-Alb/Glu | >4.3 | 78.8 (61.1 - 91.0) | 100 (63.1 - 100.0) | NA | 0.21 | 0.890 (0.753-0.966) |
| PF-Alb/ADA | ≤1.2 | 93.9 (79.8 - 99.3) | 87.5 (47.3 - 99.7) | 7.52 (5.7 - 9.9) | 0.069 (0.007 - 0.7) | 0.966 (0.856-0.998) |
| PF-LDH/PF-Alb | >9.9 | 84.9 (68.1 - 94.9) | 75.0 (34.9 - 96.8) | 3.39 (2.2 - 5.2) | 0.2 (0.05 - 0.9) | 0.856 (0.711-0.946) |
| ADA/Glu | >3.8 | 93.9 (79.8 – 99.3) | 100 (63.1 - 100.0) | NA | 0.0061 | 0.989 (0.893-1) |

Table legend: The biomarkers and their ratios were compared between TPE and PE caused by CTDs. Only parameters that yielded statistically significant *P*-values <0.01 between the two groups were shown, and entered for the construction of the receiver operating characteristic (ROC) curve. The ROC curve was analyzed, and the diagnostic accuracy of the calculated cutoff value was assessed from the area under the curve (AUC) value. CI, confidence interval; LR, likelihood ratio.

**Table S5-** *Parameters that yielded statistically significant difference between malignant pleural effusion (MPE) and complicated parapneumonic effusion (CPPE).*

| Parameter | Cutoff | Sensitivity  (95% CI) (%) | Specificity  (95% CI) (%) | LR+ (95% CI) | LR- (95% CI) | AUC (95% CI) |
| --- | --- | --- | --- | --- | --- | --- |
| Blood values: | | | | | | |
| WBC, cells/μL | ≤7600.0 | 64.0 (42.5 - 82.0) | 94.7 (74.0 - 99.9) | 12.16(8.9 - 16.6) | 0.38 (0.05 - 2.7) | 0.789 (0.64-0.898) |
| CRP, mg/L | ≤87.3 | 95.2 (76.2 - 99.9) | 75.0 (47.6 - 92.7) | 3.81 (2.8 - 5.1) | 0.063 (0.008 - 0.5) | 0.899 (0.755-0.973) |
| Pleural fluid values: | | | | | | |
| Glu, mmol/L | >5.4 | 68.0 (46.5 - 85.1) | 84.2 (60.4 - 96.6) | 4.31 (3.1 - 6.0) | 0.38 (0.1 - 1.2) | 0.805 (0.658-0.909) |
| PF-LDH, U/L | ≤614.0 | 84.0 (63.9 - 95.5) | 100 (82.4 - 100) | NA | 0.16 | 0.937 (0.82-0.988) |
| ADA, U/L | ≤19.5 | 92.0 (74.0 - 99.0) | 89.5 (66.9 - 98.7) | 8.74 (7.2 - 10.6) | 0.089 (0.01 - 0.6) | 0.955 (0.846-0.995) |
| Ratios: | | | | | | |
| WBC/CRP | >144.2 | 71.4 (47.8 - 88.7) | 81.3 (54.4 - 96.0) | 3.81 (2.7 - 5.5) | 0.35 (0.1 - 1.2) | 0.818 (0.657-0.925) |
| WBC/Glu | ≤2284.6 | 92.0 (74.0 - 99.0) | 79.0 (54.4 - 93.9) | 4.37 (3.4 - 5.7) | 0.10 (0.02 - 0.5) | 0.880 (0.747-0.958) |
| WBC/PF-LDH | >10.3 | 84.0 (63.9 - 95.5) | 89.5 (66.9 - 98.7) | 7.98 (6.3 - 10.0) | 0.18 (0.04 - 0.9) | 0.916 (0.792-0.978) |
| WBC/ADA | >459.6 | 92.0 (74.0 - 99.0) | 68.4 (43.4 - 87.4) | 2.91 (2.1 - 4.0) | 0.12 (0.03 - 0.5) | 0.819 (0.674-0.919) |
| CRP/Glu | ≤15.2 | 95.2 (76.2 - 99.9) | 87.5 (61.7 - 98.4) | 7.62 (6.2 - 9.4) | 0.054 (0.005 - 0.5) | 0.940 (0.81-0.992) |
| PF-LDH/Glu | ≤127.1 | 84.0 (63.9 - 95.5) | 94.7 (74.0 - 99.9) | 15.96 (13.1 - 19.5) | 0.17 (0.02 - 1.4) | 0.931 (0.812-0.985) |
| ADA/Glu | ≤3.7 | 88.0 (68.8 - 97.5) | 84.2 (60.4 - 96.6) | 5.57 (4.4 - 7.1) | 0.14 (0.03 - 0.6) | 0.933 (0.815-0.986) |

Table legend: The biomarkers and their ratios were compared between MPE and CPPE groups. Only parameters that yielded statistically significant *P*-values <0.01 between the two groups were shown, and entered for the construction of the receiver operating characteristic (ROC) curve. The ROC curve was analyzed, and the diagnostic accuracy of the calculated cutoff value was assessed from the area under the curve (AUC) value. CI, confidence interval; LR, likelihood ratio.

**Table S6-** *Parameters that yielded statistically significant difference between malignant pleural effusion (MPE) and uncomplicated parapneumonic effusion (UPPE).*

| Parameter | Cutoff | Sensitivity  (95% CI) (%) | Specificity  (95% CI) (%) | LR+ (95%CI) | LR- (95%CI) | AUC (95%CI) |
| --- | --- | --- | --- | --- | --- | --- |
| Pleural fluid values: | | | | | | |
| TP, g/dL | >40.0 | 88.0 (68.8 - 97.5) | 70.4 (49.8 - 86.2) | 2.97 (2.2 - 3.9) | 0.17 (0.05 - 0.6) | 0.788 (0.653-0.889) |
| PF-Alb, g/dL | >20.6 | 84.0 (63.9 - 95.5) | 59.3 (38.8 - 77.6) | 2.06 (1.4 - 2.9) | 0.27 (0.10 - 0.7) | 0.750 (0.611-0.86) |

Table legend: The biomarkers and their ratios were compared between MPE and UPPE groups. Only parameters that yielded statistically significant *P*-values <0.01 between the two groups were shown, and entered for the construction of the receiver operating characteristic (ROC) curve. The ROC curve was analyzed, and the diagnostic accuracy of the calculated cutoff value was assessed from the area under the curve (AUC) value. CI, confidence interval; LR, likelihood ratio.

**Table S7-** *Parameters that yielded statistically significant difference between malignant pleural effusion (MPE) and PE caused by connective tissue diseases (CTDs).*

| Parameter | Cutoff | Sensitivity  (95% CI) (%) | Specificity  (95% CI) (%) | LR+ (95%CI) | LR- (95%CI) | AUC (95%CI) |
| --- | --- | --- | --- | --- | --- | --- |
| Pleural fluid values: | | | | | | |
| PF-LDH, U/L | >206.0 | 76.0 (54.9 - 90.6) | 87.5 (47.3 - 99.7) | 6.08 (4.3 - 8.6) | 0.27 (0.04 - 2.0) | 0.895 (0.738-0.974) |

Table legend: The biomarkers and their ratios were compared between MPE and PE caused by CTDs. Only parameters that yielded statistically significant *P*-values <0.01 between the two groups were shown, and entered for the construction of the receiver operating characteristic (ROC) curve. The ROC curve was analyzed, and the diagnostic accuracy of the calculated cutoff value was assessed from the area under the curve (AUC) value. CI, confidence interval; LR, likelihood ratio.

**Table S8-** *Parameters that yielded statistically significant difference between complicated parapneumonic effusion (CPPE) and uncomplicated parapneumonic effusion (UPPE).*

| Parameter | Cutoff | Sensitivity  (95% CI) (%) | Specificity  (95% CI) (%) | LR+ (95%CI) | LR- (95%CI) | AUC (95%CI) |
| --- | --- | --- | --- | --- | --- | --- |
| Pleural fluid values: | | | | | | |
| Glu, mg/L | ≤5.4 | 84.2 (60.4 - 96.6) | 77.8 (57.7 - 91.4) | 3.79 (2.9 - 5.0) | 0.20 (0.06 - 0.7) | 0.843 (0.706-0.933) |
| PF-LDH, U/L | >586.0 | 100 (82.4 - 100.0) | 88.9 (70.8 - 97.6) | 9.00 (7.9 - 10.3) | 0 | 0.981 (0.888-1) |
| ADA, U/L | >19.5 | 89.5 (66.9 - 98.7) | 100 (87.2 - 100.0) | NA | 0.11 | 0.982 (0.892-1) |
| Ratios: | | | | | | |
| PF-LDH/Glu | >157.6 | 89.5 (66.9 - 98.7) | 96.3 (81.0 - 99.9) | 24.16 (20.4 - 28.7) | 0.11 (0.01 - 1.1) | 0.955 (0.850-0.994) |
| PF-LDH/ADA | >43.2 | 84.2 (60.4 - 96.6) | 70.4 (49.8 - 86.2) | 2.84 (2.1 - 3.9) | 0.22 (0.07 - 0.7) | 0.807 (0.664-0.908) |
| ADA/Glu | >3.6 | 84.2 (60.4 - 96.6) | 92.6 (75.7 - 99.1) | 11.37 (9.1 - 14.2) | 0.17 (0.03 - 0.9) | 0.940 (0.828-0.988) |

Table legend: The biomarkers and their ratios were compared between CPPE and UPPE groups. Only parameters that yielded statistically significant *P*-values <0.01 between the two groups were shown, and entered for the construction of the receiver operating characteristic (ROC) curve. The ROC curve was analyzed, and the diagnostic accuracy of the calculated cutoff value was assessed from the area under the curve (AUC) value. CI, confidence interval; LR, likelihood ratio.

**Table S9-** *Parameters that yielded statistically significant difference between complicated parapneumonic effusion (CPPE) and PE caused by connective tissue diseases (CTDs).*

| Parameter | Cutoff | Sensitivity  (95% CI) (%) | Specificity  (95% CI) (%) | LR+ (95%CI) | LR- (95%CI) | AUC (95%CI) |
| --- | --- | --- | --- | --- | --- | --- |
| Blood values: | | | | | | |
| WBC, cells/μL | >7800.0 | 89.5 (66.9 - 98.7) | 87.5 (47.3 - 99.7) | 7.16 (5.3 - 9.7) | 0.12 (0.01 - 1.1) | 0.891 (0.712-0.978) |
| CRP, mg/L | >19.3 | 100 (79.4 - 100.0) | 100 (59.0 - 100.0) | NA | 0 | 1 (0.852-1) |
| Pleural fluid values: | | | | | | |
| Glu, mmol/L | ≤5.2 | 79.0 (54.4 – 93.9) | 100 (63.1 - 100.0) | NA | 0.21 | 0.895 (0.716-0.979) |
| PF-LDH, U/L | >354.0 | 100 (82.4 - 100.0) | 100 (63.1 - 100.0) | NA | 0 | 1 (0.872-1) |
| ADA, U/L | >16.9 | 89.5 (66.9 - 98.7) | 87.5 (47.3 - 99.7) | 7.16 (5.3 - 9.7) | 0.12 (0.01 - 1.1) | 0.934 (0.769-0.993 |
| Ratios: | | | | | | |
| WBC/CRP | ≤228.3 | 87.5 (61.7 - 98.4) | 100 (59.0 - 100.0) | NA | 0.13 | 0.982 (0.821-1) |
| WBC/Glu | >1100.5 | 94.7 (74.0 - 99.9) | 87.5 (47.3 - 99.7) | 7.58 (5.7 - 10.1) | 0.060 (0.004 - 0.8) | 0.947 (0.787-0.997) |
| WBC/PF-LDH | ≤13.1 | 100 (82.4 - 100.0) | 100 (63.1 - 100.0) | NA | 0 | 1 (0.872-1) |
| CRP/ADA | >2.4 | 75.0 (47.6 - 92.7) | 100 (59.0 - 100.0) | NA | 0.25 | 0.911 (0.717-0.989) |
| CRP/Glu | >3.4 | 100 (79.4 - 100.0) | 100 (59.0 - 100.0) | NA | 0 | 1 (0.852-1) |
| PF-LDH/Glu | >59.4 | 100 (82.4 - 100.0) | 100 (63.1 - 100.0) | NA | 0 | 1 (0.872-1) |
| PF-LDH/ADA | >20.7 | 100 (82.4 - 100.0) | 87.5 (47.3 - 99.7) | 8.00 (6.2 - 10.4) | 0 | 0.882 (0.699-0.973) |
| ADA/Glu | >3.8 | 84.2 (60.4 – 96.6) | 100 (63.1 - 100.0) | NA | 0.16 | 0.954 (0.797-0.998) |

Table legend: The biomarkers and their ratios were compared between CPPE and PE caused by CTDs. Only parameters that yielded statistically significant *P*-values <0.01 between the two groups were shown, and entered for the construction of the receiver operating characteristic (ROC) curve. The ROC curve was analyzed, and the diagnostic accuracy of the calculated cutoff value was assessed from the area under the curve (AUC) value. CI, confidence interval; LR, likelihood ratio.

**Table S10-** *Parameters that yielded statistically significant difference between uncomplicated parapneumonic effusion (UPPE) and PE caused by connective tissue diseases (CTDs).*

| Parameter | Cutoff | Sensitivity  (95% CI) (%) | Specificity  (95% CI) (%) | LR+ (95%CI) | LR- (95%CI) | AUC (95%CI) |
| --- | --- | --- | --- | --- | --- | --- |
| Blood values: | | | | | | |
| CRP, mg/L | >9.7 | 85.0 (62.1 - 96.8) | 85.7 (42.1 - 99.6) | 5.95 (4.2 - 8.5) | 0.18 (0.02 - 1.4) | 0.871 (0.687-0.968) |

Table legend: The biomarkers and their ratios were compared between UPPE and PE caused by CTDs. Only parameters that yielded statistically significant *P*-values <0.01 between the two groups were shown, and entered for the construction of the receiver operating characteristic (ROC) curve. The ROC curve was analyzed, and the diagnostic accuracy of the calculated cutoff value was assessed from the area under the curve (AUC) value. CI, confidence interval; LR, likelihood ratio.
